# Solubility and Aggregation of Selected Proteins Interpreted on the Basis of Hydrophobicity Distribution

**DOI:** 10.3390/ijms22095002

**Published:** 2021-05-08

**Authors:** Magdalena Ptak-Kaczor, Mateusz Banach, Katarzyna Stapor, Piotr Fabian, Leszek Konieczny, Irena Roterman

**Affiliations:** 1 Department of Bioinformatics and Telemedicine, Jagiellonian University—Medical College, Medyczna 7, 30-688 Kraków, Poland; magdalena.ptak@uj.edu.pl (M.P.-K.); mateusz.banach@uj.edu.pl (M.B.); 2 Faculty of Physics, Astronomy and Applied Computer Science, Jagiellonian University, Łojasiewicza 11, 30-348 Kraków, Poland; 3 Institute of Computer Science, Silesian University of Technology, Akademicka 16, 44-100 Gliwice, Poland; katarzyna.stapor@polsl.pl (K.S.); piotr.fabian@polsl.pl (P.F.); 4 Chair of Medical Biochemistry—Jagiellonian University—Medical College, Kopernika 7, 31-034 Kraków, Poland; mbkoniec@cyf-kr.edu.pl

**Keywords:** solubility, complexation, hydrophobicity, antifreeze, pilin, protein structure, aggregation

## Abstract

Protein solubility is based on the compatibility of the specific protein surface with the polar aquatic environment. The exposure of polar residues to the protein surface promotes the protein’s solubility in the polar environment. The aquatic environment also influences the folding process by favoring the centralization of hydrophobic residues with the simultaneous exposure to polar residues. The degree of compatibility of the residue distribution, with the model of the concentration of hydrophobic residues in the center of the molecule, with the simultaneous exposure of polar residues is determined by the sequence of amino acids in the chain. The fuzzy oil drop model enables the quantification of the degree of compatibility of the hydrophobicity distribution observed in the protein to a form fully consistent with the Gaussian 3D function, which expresses an idealized distribution that meets the preferences of the polar water environment. The varied degrees of compatibility of the distribution observed with the idealized one allow the prediction of preferences to interactions with molecules of different polarity, including water molecules in particular. This paper analyzes a set of proteins with different levels of hydrophobicity distribution in the context of the solubility of a given protein and the possibility of complex formation.

## 1. Introduction

There are two environments of biological activity for proteins: an aquatic environment, and a membrane environment. Hence, the possibility of protein solubility is critical to protein activity. Experimental analysis of the solubility of proteins is supported by theoretical models, which is why many methods and computational tools aimed at determining the degree of solubility are available in the network [1,2]. However, the experimental techniques deliver the important basic information, including the influence of mutations [3,4,5]. The environment other than the water in the cell is the membrane environment, composed of amphiphilic molecules. The proteins anchored in the membrane can reveal the sensitivity of the polypeptide chain to its surroundings [6]. The aggregation of proteins can be treated as partial non-solubility, which is sometimes necessary in order to ensure biological activity, as well as loss of activity, as is observed in the misfolded proteins [7,8,9,10,11,12,13,14,15,16,17,18]—of which the amyloids are spectacular examples [19]. Recently, the intensively applied machine learning technique (applied in PROSO, for example) enables the prediction of protein solubility in heterologous expression in *Escherica coli* [20,21]. 

In the present study, a model called the fuzzy oil drop (FOD) [22] was used to reveal the differentiation of solubility and predisposition to the formation of the fourth-order structure, as well as the interaction of the protein with the cell membrane. The arrangement of the hydrophobicity in accordance with the proposed model justifies the high solubility of downhill- and fast-folding proteins [22]. 

The FOD model is based on the assumption that the idealized hydrophobicity distribution for a fully soluble system of bipolar molecules is the distribution expressed by the 3D Gaussian function, reflecting the hydrophobicity distribution in the spherical micelle. The values of the function spanned on the protein (sigma parameters adjusted to the size of the molecule) express the expected level of hydrophobicity at a given point. Each deviation—whether local, or covering the entire molecule of a protein or complex—identified on the basis of differences between the idealized distribution and that observed in a given protein—assesses its degree of maladjustment to the assumed distribution. The type of mismatch—local exposure of hydrophobic residues and/or local hydrophobic deficit—is interpreted either as a potential possibility of complexing another protein [23], or as the potential possibility of ligand complexation—often related to a function or even a substrate, as is the case with enzymes [24]. 

In the present study, the status of the complexes, chains, and domains present in proteins with a gradually increasing degree of mismatch in the distribution observed with the idealized one was assessed using an analysis based on the fuzzy oil drop model. The influence of the environment was demonstrated using the example of membrane proteins, where the external conditions for the part directed to the cytoplasm and the part anchored in the membrane were radically different.

The proteins were selected for analysis to represent a possibly large spectrum of different solubility and complexation tendencies [25,26,27,28,29,30,31,32,33,34,35,36]. The fuzzy oil drop model applied for analysis is based on the comparison of hydrophobicity distribution in proteins: an idealized distribution (T) (expressed by 3D Gauss function); and an observed distribution (O)—the result of inter-residual interaction [37]. Comparison of these two distributions allows for the quantitative assessment of the protein’s status and predisposition to complexation [38]. The comparison is quantitatively expressed by the parameter RD (relative distance), which measures the distance between the hydrophobicity distribution observed (O) in the protein compared to two reference distributions: T and R. T distribution represents the status with an idealized centric hydrophobic core present (T), while R represents the hydrophobicity being equally distributed all over the protein body, meaning no hydrophobic core is present. The lower the distance between O and T compared to the distance between O and R, the more expressive the presence of the hydrophobic core. In consequence, the lower RD value is attributed to the protein under consideration. The detailed description of the used model is available in [39]. 

## 2. Results

The proteins discussed here were selected taking into account the criteria of gradually developed structures: from a monomer with high solubility; through a chain with a domain structure, complexes with various forms of their stabilization, and domain-swapping; and further through complex quaternary structures with ligands present; to a multi-chain complex anchored in a membrane that acts as a channel for the transport of molecules.

### 2.1. High Solubility: Type III Antifreeze Proteins

Type III antifreeze proteins are proteins whose biological activity is related to their solubility. Their presence prevents water from taking the structural form of ice [40,41]. This work does not concern the analysis of antifreeze proteins (although they are represented in large numbers here). Their presence is related to their high solubility. These proteins are examples of proteins showing high compatibility of the O with the T distribution. This means that the surface of the protein is covered with polar groups which, by imposing a structuring of the surrounding water, prevent it from taking the structural form of ice. This imposition of the structuring of water molecules appears to be a mechanism that prevents water from freezing in organisms producing antifreeze proteins [42]. Proteins from this group were selected from short chains of monomeric form, through longer chains containing domains up to the form of a complex. The characteristics of these proteins, given by the parameter RD, are presented in Table 1.

Type II and III antifreeze proteins represent structures with very low RD values. Here, an example of this group of proteins is a 66-amino-acid chain in a fish antifreeze protein from *zoarces viviparus*—zvafp13 (PDB ID 4UR4). The O distribution in this protein is very close to that of the T distribution, which guarantees the high solubility of this protein. It should be noted that the compatibility of the O distribution with the T distribution indicates the presence of a centric concentration of hydrophobicity with the presence of the polar surface of the protein. Similarly, the double-length protein—the Ca^2+^-dependent type II antifreeze protein (PDB ID 6JK4)—also shows a distribution matching the idealized one. This agreement indicates exposure of polar residues to the surface. 

The dimeric structural form of type III fish antifreeze protein from *zoarces viviparus*—zvafp6 (PDB ID 4UR6)—deposited in the PDB does not appear to be the form found in nature. The monomer status shows very low RD values, which means full surface coverage by polar residues. The globular (close to spherical) structure of each chain of this protein is accompanied by very little intermolecular contact. Intermolecular contact relies mainly on several interactions, based on the interactions of polar groups. The impact of the electrostatic type in the aquatic environment, in the form of limited contact, is highly unlikely. At the same time, a very low RD value for single structures indicates the full availability of the surface for interactions with water.

**Figure 1:**
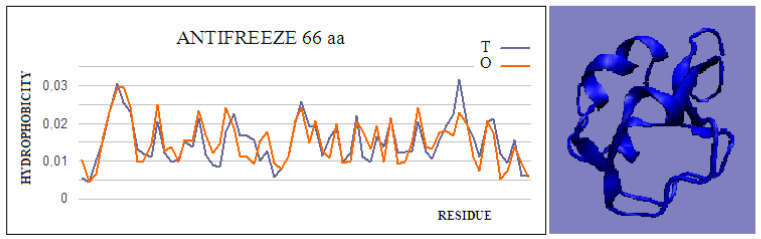
Hydrophobicity profiles. T: idealized (blue); O: observed (red), together with 3D presentation showing the highly globular form of this protein (PDB ID 4UR4). The program VMD was used to present the 3D form http://www.ks.uiuc.edu/Research/vmd/, accessed on 15 March 2021.

**Figure 2:**
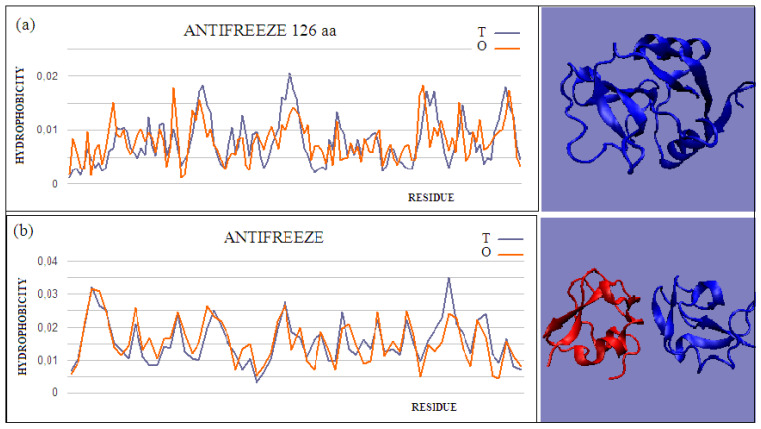
Two examples of proteins with high T and O compatibility of longer chains: (**a**) PDB ID 6JK4, and complex form (**b**) PDB ID 4UR6, together with 3D presentation, respectively. The program VMD was used to present the 3D form http://www.ks.uiuc.edu/Research/vmd/, accessed on 15 March 2021.

**Figure 3:**
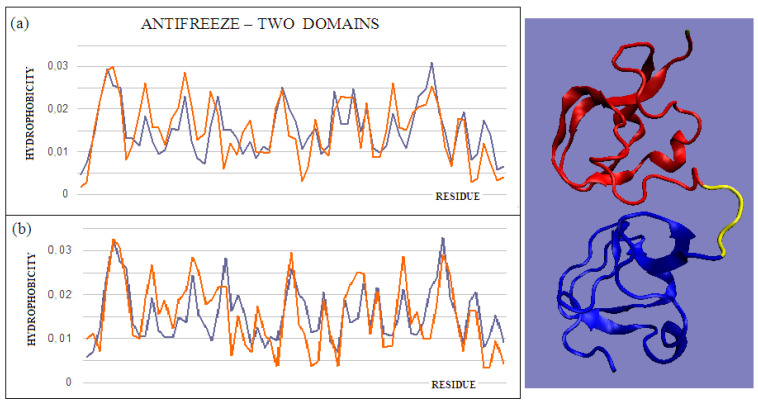
T and O profiles of an antifreeze protein with a two-domain structure. (**a**) N-terminal domain, and (**b**) C-terminal domain with a 3D presentation of this protein. Color-distinguished domains—yellow fragment: linker with high structural freedom (PDB ID 1C8A). The program VMD was used to present the 3D form http://www.ks.uiuc.edu/Research/vmd/, accessed on 15 March 2021.

The intramolecular dimer antifreeze protein rd3 (PDB ID 1C8A) has an RD value of >0.5 (Figure 1). The structure of this chain consists of two domains that are almost completely isolated from one another, linked only by the segment 55–72 in the form of a loose loop. This loop likely shows significant structural variation, which may result in the free movement of domains with a clearly polar surface. The imposition of a structure different from that of ice on the surrounding water molecules is apparent here. The high polarity of the protein’s surface guarantees its high solubility, without which its biological function of resisting the structuring of water molecules into the form of ice would be impossible. Figure 2 and Figure 3 show the high compatibility of the O and T distributions in the proteins under discussion.

### 2.2. Domain Swapping as a Way to Build Complexes

**Figure 4:**
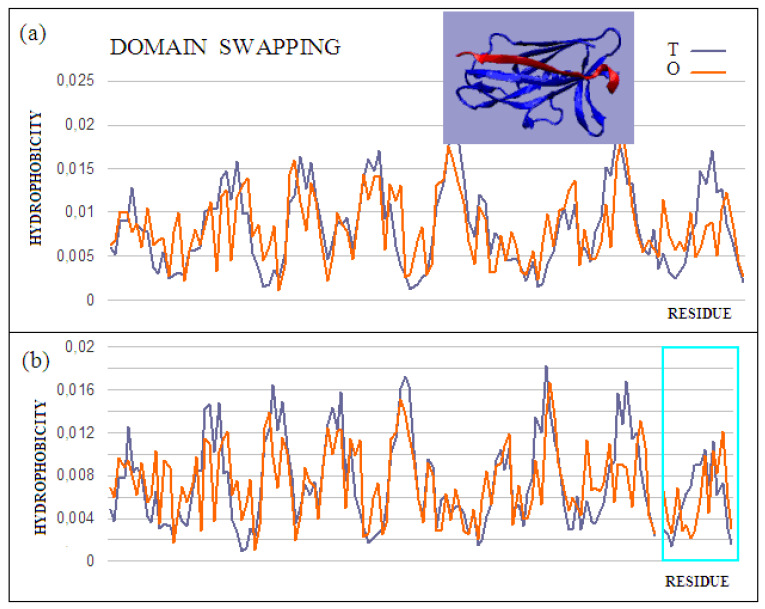
Characteristics of the domain-swapping complex present in an outer membrane protein (PDB ID 2CO2). (**a**) T and O profiles for chain A, and (**b**) T and O profiles for chain A with chain B incorporated. Turquoise frame: chain B; Inset: 3D structure; –red: chain B. The program VMD was used to present the 3D form http://www.ks.uiuc.edu/Research/vmd/, accessed on 15 March 2021.

The domain-swapping phenomenon is the exchange of structural elements between proteins, resulting in the formation of dimeric (or polymeric) forms. A chain fragment of one protein interacts with another protein in order to complete its super-secondary structure. This fragment, leaving its position in the native protein, opens the site for complexation of a fragment of the chain of another protein. The reaction can cause a domino effect. The domain-swapping phenomenon is observed relatively often [43,44,45]. The answer to the question about the mechanism of this phenomenon, based on the fuzzy oil drop model, shows that the structures of the hydrophobic core complement one another. Two sample proteins will be discussed using the fuzzy oil drop model. One is limited to docking a short chain that conforms to a slightly deformed beta-barrel or beta-sandwich domain arrangement (PDB ID 2CO2) (Figure 4). In the second case, the replacement of chain sections concerns the chain’s short fragments. The structure of the domain *Salmonella enterica* SafA pilin in complex with a 19-residue SafA Nte peptide shows the status of the idealized distribution. This means that it can function as an independent structural unit in the aquatic environment. Complexation of the 16-amino-acid polypeptide chain, however, results in an additional reduction in the RD value, which means that the status of the complex is more favorable for the structure as a whole.

This is also evidenced by the change in the status of the system itself: a slightly deformed beta-barrel shows an RD of 0.428, and in the composition extended by the presence of an additional polypeptide shows an RD of 0.416. Similarly, the status of the adjacent segments to the incorporated peptide shows an RD of 0.664, while the status of the same system in the presence of a polypeptide decreases to an RD of 0.533. The presence of the polypeptide in each of the considered systems turns out to be advantageous in reducing the RD value, which means obtaining a distribution close to the idealized distribution. This, in turn, favors obtaining a micelle-like structure, which is a favorable system in the water environment for bi-polar molecules, with different degrees of differentiation of the polar to the hydrophobic parts. Thus, a micelle-like structure is the optimal solution here. The status of the components of this complex is shown in Table 2.

The core pilin domain (PDB ID 3CRF) structure is more complex. It consists of a system of two chains (A, B) and a C peptide. The B domain provides fragments 0–19 of the A domain. However, the B domain also includes the C 0–19 peptide. The structure of the beta-structural system forms a deformed beta-barrel. Fragments 0–19 are part of these deformed beta-barrels. The complex does not exhibit an ordered structure of the fuzzy oil drop model. This is mainly due to the absence of a common hydrophobic core. In a central part of the complex, where the hydrophobic core is expected, the loosely packed interface is present. Chains A and B, treated as individual structural units, show a status minimally exceeding the adopted threshold level (RD = 0.5). Connecting fragments 1–19 results in the reduction of the RD value to 0.500 (Table 2). In the given examples, the presence of an incorporated chain fragment from another protein molecule results in a lower RD value, which means a better adjustment to an idealized state.

### 2.3. Protein in the Form of a Four-Chain Complex with a Clearly Defined Biological Function

Why is the four-chain protein with a high RD value the example for protein solubility considerations? This question will be answered via the analysis of the structure of human hemoglobin (PDB ID 1A3N). According to the fuzzy oil drop model, protein solubility is represented by the protein’s RD being less than 0.5. This value means that the surface of the protein is covered with polar groups with centrally concentrated hydrophobic residues. The full agreement of the O and T distributions expresses a very high solubility, eliminating any interaction with other molecules except water, ions, or other molecules of a polar nature. The presence of a biological function requires exceptions to this rule. The specificity of the local deviation determines the specificity of the biological activity. The exposure to hydrophobic residues on the surface implies the formation of protein–protein complexes. Contact with hydrophobic surfaces eliminates the unfavorable—in terms of entropy, contact with water. Identification of local exposure suggests the complexation site of another protein, or binding to a bipolar ligand. The local hydrophobicity deficit, often accompanying the presence of cavity, suggests a site of interaction with the ligand or substrate. Therefore, the identification of the part of the protein involved in the complexation of a ligand or another protein allows for the prediction of the site of complexation. The fragments of the polypeptide chain eliminated from the RD analysis make it possible to identify that part of the protein, the hydrophobicity arrangement of which is consistent with the assumed model, and thus indicates the locations of the parts of the protein responsible for its solubility. Such operations were performed for hemoglobin. The RD parameter values for the four-chain complex show high mismatch. The status of individual chains shows compatibility with an idealized distribution (in the case of a chain A, near compatible) (Table 3).

A comparison of RD values for the complex and the individual chains indicates:

1. The structure of individual chains is close to the idealized distribution;
2. The presence of a ligand-binding cavity affects the incompatibility of the O and T distributions;
3. The difference between the α and β chain characteristics, from the point of view of both P–P interaction and ligand binding; and
4. The dominant role of the interface structure on the complex stabilization, with the presence of the ligand-binding cavity having a much smaller effect.

**Figure 5:**
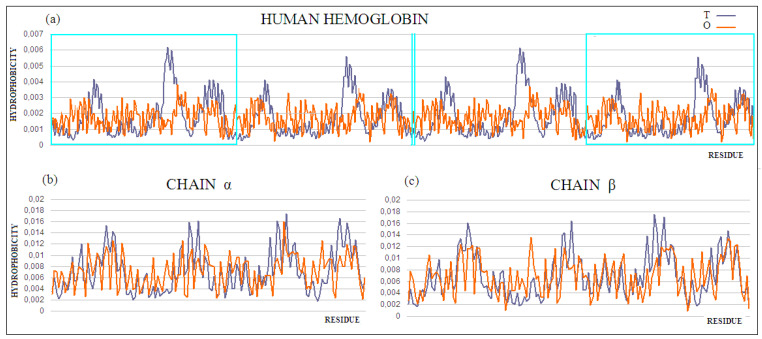
(**a**) T and O profiles for the quaternary structure of human hemoglobin. Status of the (**b**) α and (**c**) β chains was determined for these chains as independent structural units. The α and β chains are marked on profile (**a**) with turquoise frames, and shown in (**b**) and (**c**), respectively.

**Figure 6:**
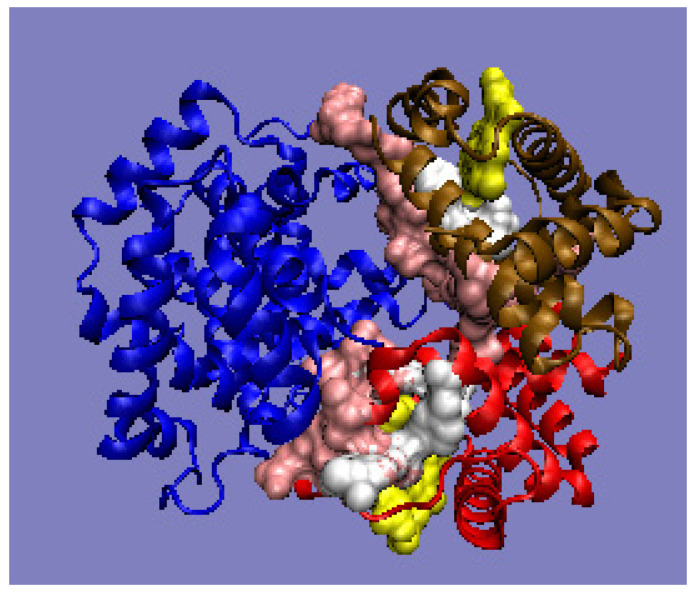
3D presentation of human hemoglobin: chain α (red), and chain β (magenta). –White: heme particles; pink: residues involved in the P–P interaction; yellow: residues involved in ligand binding. The purpose of the highlighting (pink and yellow) is to indicate the position of those residues which cause the RD parameter to be increased. The program VMD was used to present the 3D form http://www.ks.uiuc.edu/Research/vmd/, accessed on 15 March 2021.

The comparison of the RD values suggests the differentiation of the characteristics of A chains versus the B chain, which is observed in the experimental studies [46,47]. The operation, consisting of the gradual elimination of the residues showing the highest deviations of the O distribution from the T distribution for the complex, leads to the identification of the part representing the best fit to the system, compatible with the fuzzy oil drop model, ensuring the solubility of hemoglobin. This is visualized by the profiles (Figure 5) and the 3D structure (Figure 6), with the marked residuals causing an increase in the RD value. Their elimination (in the calculation of D_KL_) results in obtaining the status expressed as RD < 0.5.

The analysis of the results also suggests the presence of distortions in the hydrophobicity distribution resulting from the presence of the ligand-binding cavity. The identified part (after the elimination of residues showing large differences between the T and O distributions) allows for the extraction of the part of the complex that, showing the order proper to the aqueous environment, ensures the solubility of the complex. This indicates the presence of local inconsistencies with the idealized distribution, in the form of a local hydrophobicity deficit (in the case of the cavity) and a local excess (in the case of interaction with other chains). Eliminating the residues showing these mismatches from the computation results in an RD of <0.5. This part of the complex is responsible for the micelle-like organization and, therefore, also for solubility.

### 2.4. Complex Stabilized by an Additional Domain in the Interface Region

**Figure 7:**
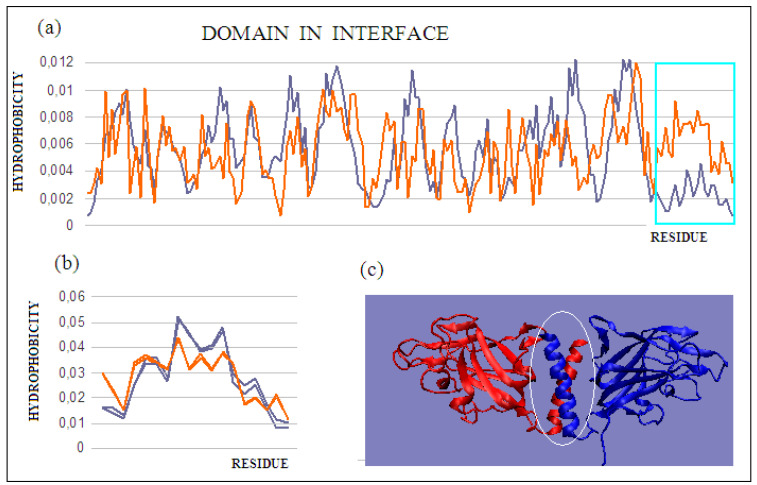
T and O distributions for: (**a**) a single chain with the C-terminal fragment present, showing a clear mismatch with the structure of the chain as a structural unit, and (**b**) two superimposed C-terminal fragments (helices) constituting an independent common domain showing high micelle-like ordering; (**c**) 3D presentation with a marked domain present in the interface. Chains are of various colors. The program VMD was used to present the 3D form http://www.ks.uiuc.edu/Research/vmd/, accessed on 15 March 2021.

The durability of the complex can be achieved by generating an additional domain in the interface area. Such a domain, which consists of fragments derived from both complexed chains and dimers, is an example of synergy in attaining the goal of complex stability. Such a solution was identified earlier in dystrophin [48]. The presence of such a domain in the interface, in the case of dystrophin, allows for significant deformations (external stresses), ensuring a return to the initial state after the external forces have subsided. In the present review of methods for stabilizing complexes, the example of such an approach is neuropilin-2 (PDB ID 4QDS). The researchers who supplied the structure to the PDB describe the discussed system as a stable dimer [32]. The dimer status is expressed by an RD value of 0.716 (Figure 7a), which indicates a far from stable hydrophobicity distribution with a centric core. Based only on the value of this parameter, it can be concluded that the complex is not stable. The status of the chains treated as individual structural units for the sections (272–453) shows a significant maladjustment in the C-terminal sections of both chains (Figure 7b (Table 4)). Their elimination, and the determination of the T and O distributions for the A chain (272–434), reveals a much better fit of the T and O distributions (Figure 7c). On the other hand, the generated new domain—not identified in any monomer—which includes C-terminal fragments of monomer chains, represents the status defined by an RD value of 0.419 (Figure 7c). The presence of such a durable structural element in the complex guarantees its indissolubility. The common domain, present in this domain’s most stable interface, with a clearly generated centric core and a surface providing entropy-favorable contact with water, guarantees the stability of the complex.

The protein discussed here is an example of a complex formation mechanism involving chain sections not only in interface creation, but also in the structuring of a new separate domain that fully functions as an interface. 

### 2.5. Multi-Chain Complex: A Pilin

An exemplary object for the analysis of a multi-chain complex is pilin [33,34]. This complex was selected because it combines the properties of the complex with the biological function of this protein, which is the transport of molecules (genetic material). The biological function also requires the complex to be anchored in the cell membrane, and the channel must be present in the central part of the elongated structure. The structure of the monomer, its fragments, and the final structure of the functional complex were analyzed. The complex must be anchored in the cell membrane in order for the canal to function. This requires the presence of a hydrophobic part in contact with the environment of the membrane—an area with a structure inconsistent with the model used. Based on the fuzzy oil drop model, it is also expected that the entire complex structure should show the absence of a hydrophobic centric core due to the presence of a channel. The distribution of the mentioned properties should additionally change with the movement from the outer pole to the pole anchored in the membrane. The analysis of this complex began with the presentation of the monomeric molecule (Table 5).

**Figure 8:**
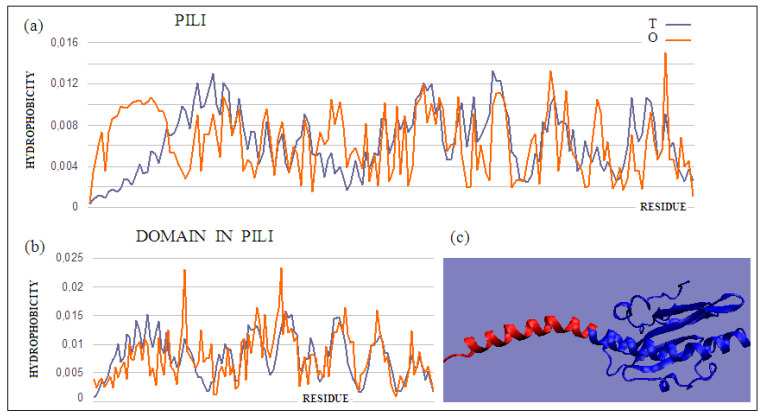
T and O distributions for (**a**) a complete molecule showing a significant mismatch of the distribution expected in the N-terminal helix, and (**b**) the globular part of the molecule (navy blue part shown in (**c**)); (**c**) 3D presentation of the discussed monomeric pilin molecule. A fragment of the N-terminal helix (red) and a globular domain (blue) were distinguished. The program VMD was used to present the 3D form http://www.ks.uiuc.edu/Research/vmd/, accessed on 15 March 2021.

The pilin monomer molecule (PDB ID 2PIL) consists of a long helix in the N-terminal part of the chain and a globular part (PDB ID 1HPW) (Figure 8). Analysis of the globular part shows the structure with a low RD, while the complete molecule already shows an RD value above the adopted threshold. This high value is due to the significant maladjustment present within the helix, which is apparent due to the exposure of the helix and its low packing, deviating from globularity in this part of the molecule. Analysis of the T and O profiles for this molecule shows a significant excess of hydrophobicity in the area where the T distribution suggests low O values. This fragment of the chain is the N-terminal part of the helix, which in the second half of its length represents the matching distribution expected as part of the packed domain.

**Figure 9:**
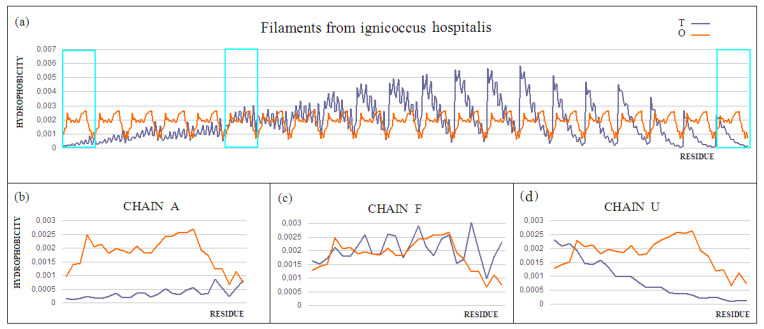
T and O profiles for the status of (**a**) a complex consisting of 21 chains—those monomeric units that differ significantly in status throughout the complex are distinguished, (**b**) chain A, anchored partly in membrane, (**c**) chain F, located in the central part of the complex, showing a fairly close agreement of the T and O distributions, and (**d**) chain U, the outer chain.

The mismatch in the status of the helical section, which does not integrate into the globular section at high O values, must have a purpose. This is evident from the analysis of the final pilin complex. The compatibility of the T and O distributions within the globular domain prepares this part of the final complex for favorable contact with the aqueous environment. A clear excess of hydrophobicity in the N-terminal helix suggests its use in building a part prepared for interaction with the cell membrane. The helices extracted from the full complex and included in filaments from *ignicoccus hospitalis* show diversity of packing in proteins containing N-terminal type IV pilin helices, arranged conically to form a geometrically ordered system with an axial structure in the presence of symmetry (PDB ID 3J1R) (Figure 9a). 

The status of this complex is expressed by a very high RD value (see Table 5).

**Figure 10:**
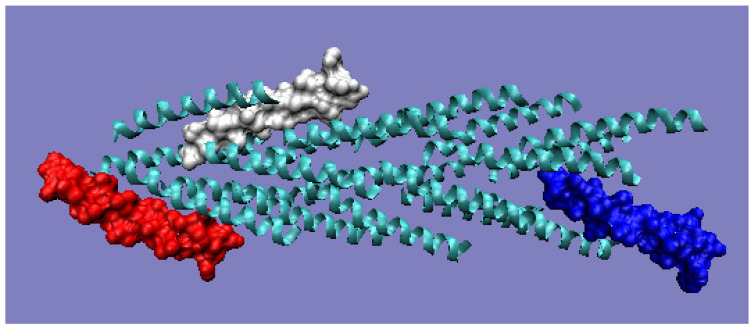
3D presentation of filaments from *ignicoccus hospitalis* with highlighted monomers, the status of which is shown in the T and O profiles (Figure 9). Red: chain A; white: chain F; blue: chain U. The program VMD was used to present the 3D form http://www.ks.uiuc.edu/Research/vmd/, accessed on 15 March 2021.

Chain A exhibits a significant excess of hydrophobicity in the complex, coming from not one but several surrounding helices derived from adjacent monomers. This is closely related to the need to anchor the complex in the cell membrane—i.e., in a highly hydrophobic environment (Figure 9b and Figure 10). Chain F was seen (Figure 9c and Figure 10) to show an arrangement close to that of a micelle-like construction. Similarly, the chain U helix exhibits a fairly large excess of hydrophobicity due to its nature (Figure 9d and Figure 10). It seems that the structure consisting solely of the helices constituting the piles multiplies redundancy in the N- and C-terminal parts, while the central part of the complex shows a significant deficit of hydrophobicity relative to the assumed hydrophobicity concentration in the form of a hydrophobic core, which is not present here. 

**Figure 11:**
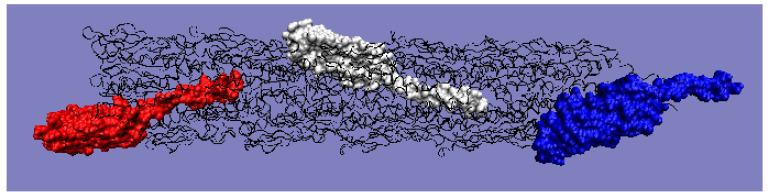
3D structure with color-highlighted chains. Red: chain A; white: chain K; blue: chain U. The program VMD was used to present the 3D form http://www.ks.uiuc.edu/Research/vmd/, accessed on 15 March 2021.

**Figure 12:**
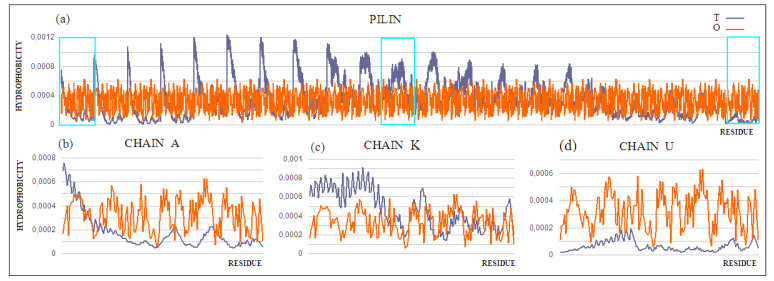
T and O profiles for (**a**) the chains present in the pilin. 3D Gaussian function generated for the entire complex. Three strings shown individually in (**b**–**d**) are marked: (**b**) chain A—outer; (**c**) chain K—central, and (**d**) chain U—membrane.

Moving on to the analysis of the complete pilin complex (PDB ID 5VXY) (Figure 11) composed of complete chains, it should be noted that there is an obvious inconsistency of the T and O distributions resulting from the elongated and non-globular structure of the complex (Figure 12a). The analysis of the O profile shows an almost uniform distribution of hydrophobicity along the entire complex, with no differentiation between the individual monomers. The analysis of the individual chains (treated as components of the complex—the 3D Gaussian function generated for the entire complex) reveals the preparation of the pilin complex for interaction with the cell membrane (Figure 12b–d). This reveals the status of chain U—the membrane-anchored end—showing significant local redundancy of hydrophobicity. Chain K reveals (Figure 12c) a relatively high correspondence, especially with regard to the status of the helix, while chain A—the outer part—shows high hydrophobicity in the “embedded” part of the complex. It should be remembered that the globular domain (Figure 12a), even locally, shows an adaptation of the T and O distributions to the micelle-like system. Closer analysis shows a common tendency in the form of the expected high level within the helices (N-thermal chain fragment), which are responsible for anchoring in the cell membrane (chain U), as well as the formation of a stable inter-chain interaction within the complex itself (Figure 12d).

Chain K, in the central part of the complex, shows the closest approximation of the T and O distributions, but this convergence is only due to the fact that it is located appropriately in relation to the assumed 3D Gaussian distribution.

The pilin complex as a channel (apart from anchoring in the membrane), and thus representing a system with a fairly high structural and functional complexity, requires in-depth analysis. This complex is an object of independent analysis. Here, only the high readability of the analysis, based on the fuzzy oil drop model, in relation to the prediction of the solubility or complexity of the molecules in question, was demonstrated. A separate issue is the assessment of the status of a single chain in such a composite complex as pilin. All of the monomers in the complex showing the same sequence get different roles to play within the complex. Table 5 highlights these status changes. The globular (beta-structure) part of the monomer shows a very high ordering according to the distribution of the soluble molecule (column Mon—from monomer). In the environment of the complex, this status changes, and remains the most similar in the exposed part (chain A)—the part in contact with the aquatic environment. The elimination of the P–P contact residues lowers the RD value of the monomers, which means that exposure to the hydrophobic residues at the surface occurs, and prepares it for interaction with another chain.

## 3. Discussion

The mechanism of creating quaternary structures is very diverse, and depends on the desired biological function. Usually, the method of obtaining two- or multi-chain complexes has one goal: to ensure specific biological activity. Nevertheless, the means of achieving these goals can vary. Obtaining highly complex systems cannot be simple and similar for many different systems. The examples presented here provide one amongst many possible mechanisms. The analysis presented here is intended as a background reference for the study of the formation of misfolding complexes. The repeatability of O versus T profiles in the case of, for example, pilin—a multicomponent fibril—helps to identify the specificity of amyloid structures, where the repeatability of hydrophobicity distributions is also very high. 

There is a question as to why amyloid propagates indefinitely, while piline has a defined, limited, and repeatable length (limited number of chains in the complex). The solutions presented here do not exhaust the spectrum of possibilities leading to the formation of quaternary structures. Nevertheless, it is impossible to eliminate the aquatic environment as a factor which may play a decisive and active role in the complexation process. Antifreeze and hemoglobin proteins present in the analyzed collection act as reference structures. Antifreeze proteins show high solubility, representing a high degree of similarity to the T and O distributions, which indicates the concentration of hydrophobic residues in the center of the molecule, with the exposure to polar residues on the surface. As a result, the surface is covered with polar systems that constitute objects for beneficial interaction with water molecules in the environment. The presence of hemoglobin results from the need to demonstrate the presence of inter-chain complexation and the complexation of large ligands. The presence of ligands is closely related to the performance of biological activity. The structure of hemoglobin, despite significant deviations of the O distribution from the T distribution resulting from the biological function performed, shows the presence of some chains that meet the criteria qualifying them as guaranteeing solubility. The analysis of the multi-chain association with a fibrillary structure can be an example for comparisons with the structures of amyloid fibrils. The lack of a hydrophobic core in both cases is common, expressed by the deviation of the O distribution from the T distribution. The main difference, however, lies in the maintenance of at least some of the globular form of the chains constituting the canal, while the amyloid chains show a 2D Gaussian structure, representing flat structures. The consequence is that all residues present are involved in the inter-chain interactions in amyloids, whereas in pilin this occurs only for amino acids representing the appropriate status to the rest of the molecule. The 2D Gaussian form promotes and even guarantees unlimited growth of the fibril. 

A review of proteins with diversified structures and biological activities, in terms of the increasing amount of information carried by a given protein and the degree of complexity of its structure, is discussed in detail in [22,23]. The summary of this analysis reveals the participation of the water environment in the construction of the quaternary structure. The phenomenon of solubility measured by the parameter expressing the degree of hydrophobicity in the micelle-like structure was considered. The quantified presence of surfaces with exposed polar systems is present, in addition to deviations from this type of ordering resulting from the specific biological activity of the protein or complex. Biological activity in the form of interaction with specific chemical compounds requires the presence of an equally specific disturbance of the micelle-like structure. The exposure of hydrophobic residues to the surface is used to complex another protein. 

The formation of the multi-molecule system as a channel is similar to the observation of amyloids in the form of a cyclically repeated deviation in the distribution observed in the protein versus an idealized distribution. The analysis carried out here is one approach to determining the differences that enable the identification of amyloid complexing versus other fibrillar forms of multi-molecular complexes. This differentiation is based on the presence of 3D Gaussian globular structures in the case of single chains included in functional complexes, while amyloid fibrillary complexes represent a form with 2D Gaussian distribution with respect to a single chain of the fibril component. This means that biologically active protein complexes are constructed from globular units (domains, chains), while in the case of amyloids, specific flat surfaces in the form of successive patches are observed.

The present analysis is focused on the complexation of small proteins. The problems of the relationship between the size of monomer interfaces in oligomers, as well as the identification of large hydrophobic cavities in proteins, are the main issues under consideration in the current analysis [49].

## 4. Materials and Methods

### 4.1. Data 

The proteins were selected for analysis to represent possibly highly different proteins with respect to their solubility and complexation (Table 6). 

The set of selected proteins is presented in Table 1, where proteins showing variability in both solubility and complexation with other proteins, as well as the environment of their activity in the cell, are included.

### 4.2. The Fuzzy Oil Drop Model 

The applied model (fuzzy oil drop) assumes the hydrophobicity distribution in the form expressed by the 3D Gaussian function. This function reflects the distribution of hydrophobicity with a centric concentration of hydrophobicity, and its gradual reduction with distance from the center, reaching values close to zero on the protein surface. The size of the ellipsoid expressed by this function depends on the size of the analyzed molecule. This function, spread over the protein molecule, is defined by the values of the parameters σX, σY, and σZ, determined according to the size of the protein. The level of idealized hydrophobicity for a given residue (Ti) expresses the value of the 3D Gaussian function in the position of the so-called effective atom (the average position of the atoms making up a given amino acid). This idealized distribution is confronted with the observed distribution (O), which is the result of hydrophobic interactions between the residues. This value depends on the distance between the effective atoms, and on the intrinsic hydrophobicity of the interacting amino acids [37]. The degree of similarity between the T and O distributions is assessed based on the divergence of entropy (D_KL_) [38].

The second reference distribution against which the O distribution is assessed is the R distribution, which is the unified distribution, where each residue (effective atom) is assigned a constant hydrophobicity level equal to 1/N, where N is equal to the number of amino acids in the chain. Comparing the distance between the O and T distributions with the distance between the O and R distributions enables us to determine the similarity of the O distribution to each of the reference distributions. The lower distance from the O distribution to the T distribution is interpreted as the presence of a hydrophobic core in the analyzed protein. It is expressed by the parameter RD (Relative Distance). RD is equal to the ratio of the O–T distances to the sum of the O–T and O–R distances. The O–T distance is the value of D_KL_ for the O distribution, taking the T distribution as the reference distribution, while the O–R distance expresses the D_KL_ for the O distribution, taking the R distribution as the reference distribution. An RD value of less than 0.5 indicates the presence of a hydrophobic core. The concept of a hydrophobic core, apart from the concentration of hydrophobicity in the center of the molecule, also expresses the presence of a polar surface. Identified amino acids showing a deviation between the T and O distributions are analyzed for their contribution to a biological activity. This model has been described many times [39].

## 5. Conclusions

The paper demonstrates the possibility of quantifying environmental preference for protein solubility using the fuzzy oil drop model. The diverse nature of the proteins in question reveals the consistency of the measure expressed by the RD parameter, with the presence of a polar surface guaranteeing solubility with the presence of local disorders in the distribution of hydrophobicity observed in the investigated protein against an idealized distribution. These local variations of high specificity determine the specificity of the biological activity of a given protein. The use of the FOD model makes it possible to assess the status of the chain in relation to the hydrophobic environment of the membrane proteins. Exposure to the hydrophobic surface identified in the protein structure is a factor that promotes interaction with the membrane. The model used introduces the possibility of quantifying this specificity. The present analysis recognizes the conditions that guarantee the solubility of proteins against the phenomenon of amyloid insolubility. In the search for differences between amyloids and complex proteins with biological activity, the solubility phenomenon is of critical importance. The analysis of the list of proteins with different characteristics, including the membrane protein, reveals a whole spectrum of possibilities of identifying the types and the degree of differentness of characteristics in relation to the aquatic and non-aqueous environments expressed by the fuzzy oil drop model.

## Figures and Tables

**Table 1:** RD parameters for representatives of the soluble protein group. This group includes type III antifreeze proteins of various structures: single chains of varying lengths, a two-domain protein, and a dimer.

PDB ID	Characteristics	RD—Complete Chain	Selected Fragments	RD
4UR4	66 aa	0.289		
6JK4	126 aa	0.489		
4UR6	64 aa—dimer	0.638	Chain A Chain B	0.298 0.280
1C8A	134 aa—two domains	0.659	Dom1 Dom2	0.293 0.280

**Table 2:** A set of values of the RD parameter for structures created via domain swapping.

PDB ID	Target Mol.	RD	Incorporated	RD	Complex	RD
2CO2	Pilin domain, 48–170	0.472	N-terminal 27-45	0.725	Complex	0.456
	Beta-barrel	0.428			Beta-barrel with beta-fragment incorporated	0.416
3CRF	Chain A	0.509	Chain C	0.731	Complex A+C	0.500
2J6R	Chain A	0.669			Chain A(no 24-31) with B(24-31)	0.662

**Table 3:** The RD value parameters for the four-chain hemoglobin complex and the individual chains. The chain A and chain B positions indicate the status of chains treated as individual structural units, with 3D Gauss function generated for each of them individually. The P–P position indicates the status of the residues involved in the interface. The No P–P position indicates the part of the molecule with excluded residues involved in the complexation of another chain. The Ligand position indicates the status of residues involved in ligand binding. The No Lig position indicates the status of the rest of the chain, excluding the residues involved in ligand binding.

Form	RD				
		P–P	No P–P	Ligand	No Lig
Complex	0.726	0.718	0.685	0.748	0.722
Chain A	0.506	0.501	0.526	0.633	0.491
Chain B	0.427	0.500	0.424	0.552	0.398

**Table 4:** RD values for the complex of two chains that generate an additional common domain in the interface made up of parts of the monomer chains.

Structural Unit	Fragment	RD
Dimer		0.716
Chain A in dimer		0.702
Chain B in dimer		0.729
Chain A—individual	272–452	0.614
Chain B—individual	272–452	0.645
Chain A—individual	272–433	0.510
Chain B—individual	272–433	0.474
Helices in new domain	434–451 A+B	0.419

**Table 5:** Status of single chains, chain fragments, and the pilin complex. P–P: the status of the residues involved in the protein–protein interaction; No P–P: the remaining part of the molecule/chain after elimination of the residues involved in P–P; Beta, Helix: the status of fragments representing a given type of secondary structure determined within the monomer (Mon) and complex (Comp).

Monomer				
PDB ID	Chain	Beta-Sheet	Helix	
1HPW	0.422	0.350	0.478	
2PIL	0.609	0.392	0.811	
**Complex**				
	**Complex**	**Mon/Compl**	**Beta Mon/Comp**	**Helix Mon/Comp**
3J1R	0.873	0.601/0.781 A /0.482 F /0.884 U		
5VXY	0.803	0.645/0.740 A /0.779 F /0.700 U	0.231/0.322 /0.546 /0.507	0.838/0.745 A /0.683 F /0.784 U
		**P–P**	**No P–P**	
5VXY	0.803	0.736/0.605 A /0.679 F /0.720 U	0.752/0.523 A /0.663 F /0.538 U	

**Table 6:** The set of proteins analyzed in the present work, together with their short characteristics.

PDB ID	Characteristics	Biological Function	Criterion of Qualification	Reference
4UR4	66 aa	Antifreeze type III	High solubility	[25]
6JK4	126 aa	Type II		[26]
4UR6	64 aa—dimer	Type III	Dimer	[25]
1C8A	134 aa—two domains	Type III	Two domains	[27]
1Y7Q	Dimer	C-terminal domain (CTD) of the capsid protein	P–P interaction	[28]
2CO2	Domain swapping	Outer membrane protein	Domain swapping	[29]
3CRF	Domain swapping	Outer membrane protein	Domain swapping	[30]
1A3N	Complex + ligand binding	Hemoglobin	Quaternary structure ligand	[31]
4QDS	Domain in interface	Cell adhesion	Domain in interface	[32]
2PIL	Unit in IV pilus	Pilus	A unit of a multi-molecular complex	[33]
1HPW	Globular part IV pilus	IV pilus	Globular part	[34]
3J1R	Filaments N-terminal type IV pilin helices	Type IV pilin	Channel’s elements	[35]
5VXY	Pilin	Channel for genetic material transport	Fibril structure of 21 chains with a channel transporting molecules	[36]

## Data Availability

All data are available from the corresponding author(s) on request.
